# Higher lactate production from glucose in cultured adipose nucleated stromal cells than for rat adipocytes

**DOI:** 10.1080/21623945.2019.1569448

**Published:** 2019-02-08

**Authors:** Floriana Rotondo, Ana-Cecilia Ho-Palma, María del Mar Romero, Xavier Remesar, José Antonio Fernández-López, Marià Alemany

**Affiliations:** a Department of Biochemistry and Molecular Biomedicine, Faculty of Biology University of Barcelona, Barcelona, Spain; b Institute of Biomedicine, University of Barcelona, Barcelona, Spain; c CIBER-OBN Research Web, Barcelona, Spain

**Keywords:** Adipose tissue, glycolysis, lactate, glycerol, adipocytes, stromal cells, glycaemia

## Abstract

White adipose tissue (WAT) nucleated stromal cells (NSC) play important roles in regulation, defense, regeneration and metabolic control. In WAT sites, the proportions and functions of NSC change under diverse physiological or pathologic conditions. We had previously observed the massive anaerobic wasting of glucose to lactate and glycerol in rat epididymal adipocytes. To test site variability, and whether the adipocyte extensive anaerobic metabolism of glucose was found in NSC, we analyzed, in parallel, subcutaneous, mesenteric and epididymal WAT of male adult Wistar rats. Adipocytes and NSC fractions, were isolated, counted and incubated (as well as red blood cells: RBC) with glucose, and their ability to use glucose and produce lactate, glycerol, and free fatty acids was measured. Results were computed taking into account the number of cells present in WAT samples. Cell numbers were found in proportions close to 1:13:100 (respectively, for adipocytes, NSC and RBC) but their volumes followed a reversed pattern: 7,500:10:1. When counting only non-fat cell volumes, the ratios changed dramatically to 100:10:1. RBC contribution to lactate production was practically insignificant. In most samples, NSC produced more lactate than adipocytes did, but only adipocytes secreted glycerol (and fatty acids in smaller amounts). Glucose consumption was also highest in NSC, especially in mesenteric WAT. The heterogeneous NSC showed a practically anaerobic metabolism (like that already observed in adipocytes). Thus, NSC quantitative production of lactate markedly contributed (i.e. more than adipocytes) to WAT global use (wasting) of glucose. We also confirmed that glucose-derived glycerol is exclusively produced by adipocytes.

## Introduction

Adipose tissue, and more especially white adipose tissue (WAT), is one of the most peculiar animal tissues. It has been proposed that its variability in site size, distribution and function is a correlate of its adaptability, diversity of functions and adaptability, constituting, as a whole, a disperse organ.^1^ After decades of assuming that WAT was largely an energy dump (of triacylglycerols, TAG), the uncovering of its multiple, complex regulatory^2^ and defense^3^ functions has increased our attention on this tissue. The direct implication of WAT in the synthesis, regulation and control of hormonal and paracrine signals,^4,5^ and their relation with inflammation^6^ clearly indicated a key role in energy metabolism and its pathologies, such as the metabolic syndrome.^7^

WAT is also implicated in defense, repair and regeneration processes.^8^ The tissue includes a large number of immune cells, such as macrophages^9^ and lymphocytes, as well as stromal endothelial cells.^10,11^ They play a key role in the control of blood flow to WAT itself^12^ and neighboring tissues.^13^ By the way, WAT (the adipose organ) *also* stores TAG and constitutes the main body energy reserve.

Mammalian WAT contains cells of three widely different types: a) adipocytes (large, with huge fat vacuoles), b) blood erythrocytes (very small, non-nucleated), and c) a wide variety of cells, such as those described above, which we opted to describe as ‘nucleated stromal cells’ or NSC, irrespective of their origin and function. This is not a systematic, but a practical experimental grouping of tissue cell fractions. NSC may be attached to adipocytes or other structures; contributing to form a fiber scaffold,^14^ or remain unattached in WAT interstitial space. The structural and functional variety of these cells is considerable, in accordance with their roles and origins, but their proportions in WAT may change because of’ exposure to external agents, environmental shock, nutrient availability and variety, inducing the activation of defense systems.^15^ The pathological importance of knowing WAT specific functions is paramount, given its mass, distribution, cell plurality, capacity to change, adapt and respond, and the dramatic consequences observed in human disease, as is the case of metabolic syndrome.^16^

In spite of the abundance of known antecedents, our knowledge of WAT metabolism remains incomplete and largely limited to a few pathways. This insufficient information is, in part, a consequence of the practical difficulties of establishing comparisons of a quite peculiar tissue, which usually contain between 50–90% fat,^17^ with as little as 1.3–1.5% of non-fat adipocyte cell volume.^18^

It is already known that mature WAT behaves, in the presence of glucose and oxygen, practically as an anaerobic tissue,^19,20^ with low oxygen consumption *in vivo*^21^ and a disproportionately high production of lactate^22^ and glycerol^23^ with respect to its mass of ‘live cytoplasm’. Primary cultures of adipocytes showed close relationships of lipogenesis, or enhanced glycolytic activity, to the size of the cells.^24^ In recent papers, we have related enzyme activities, metabolite efflux or gene expression^25-27^ data to the number of cells, but this procedure requires considerable tissue manipulation: cell isolation, counting and analysis of recovery of the cells.^28^ This necessary step undoubtedly may hamper the rapid comparative (and quantitative) analysis of WAT sites under different conditions.

Our objective was to determine, using quantitative available methods, the relative capacity of adipocytes, NSC and RBC (red blood cells) present per unit of WAT weight to use glucose, mainly to produce lactate and glycerol. Since this approach could not be done *in vivo*, we isolated the cells, and incubated them with glucose in primary cultures. Later, we “reconstructed” the WAT ability to use glucose from the number of cells and their glycolytic effectiveness in culture.

## Results

### Experimental set-up

WAT samples of weight-and age-matched male Wistar rats were pooled in two-rat samples of tissue. Samples were taken from mesenteric (MES), subcutaneous (SC; constituted by both inguinal cordons) and epididymal (EPI) WAT. Adipocytes and NSC suspensions were isolated (and compared with blood-extracted RBC) in primary culture incubations with 7 mM or 14 mM glucose during 24 h as explained under Methods. Changes in glucose, lactate, glycerol and NEFA (non-esterified fatty acids) in the medium were analyzed. Cell numbers were estimated for all samples and used in the calculations, compared with those observed in the collagenase-WAT digests.

### WAT cell proportions

The proportions (number, size) of cells (adipocytes, NSC, RBC) in the samples extracted from SC, MES and EPI WAT sites were analyzed in order to refer the experimental data to the original WAT sample, and to be able to compare and compute the results obtained from different incubation wells.

Table 1 shows the number of cells (adipocytes, NSC and RBC) present in 1 g of WAT from SC, MES or EPI WAT sites. No statistically significant differences in adipocyte or RBC numbers were observed, but in EPI WAT, the number of NSC per g was about half that found in MES, SC WAT showed values in between. No differences were found in the mean cell volume for NSC. However, MES WAT adipocytes were smaller than those of EPI WAT (had about half of their volume).

**Table 1.** WAT site cell characteristics of young male Wistar rats.

parameter	units	SC WAT	MES WAT	EPI WAT	p
cell counts					
adipocyte	cellsx10^6^/g WAT	3.19 ± 0.84	4.59 ± 0.98	2.90 ± 0.35	NS
nucleated stromal cells	cellsx10^6^/g WAT	43.8 ± 7.7	59.8 ± 3.8	27.9 ± 7.3	0.0220
red blood cells	cellsx10^6^/g WAT	108 ± 51	236 ± 57	150 ± 61	NS
cell fragments	10^6^/g WAT	151 ± 50	103 ± 22	105 ± 18	NS
WAT adipocytes recovery	%	70.4 ± 7.6	83.0 ± 8.6	77.2 ± 0.1	–
cell volumes					
adipocyte volume	pL/cell	251 ± 30	159 ± 33	309 ± 7	0.0065
nucleated stromal cells	fL/cell	419 ± 28	255 ± 79	256 ± 44	NS
red blood cells	fL/cell	32.4 ± 1.9	32.4 ± 1.9	32.4 ± 1.9	–
red blood cells	% stromal cells	62.8 ± 12.3	79.4 ± 3.3	84.4 ± 2.9	NS
cell fragments and droplets	fL/fragment	<5			–
other tissue data					
debris (dry weight)	mg/g WAT	37	59	22	–
water in intact tissue	mg/g WAT	220 ± 63	160 ± 37	64.4 ± 11.2	NS
water in tissue minus debris	mg/g WAT	213 ± 62	152 ± 41	62.9 ± 10.2	NS
fat in intact tissue	mg/g WAT	740 ± 20	744 ± 24	871 ± 12	0.0014
fat in tissue minus debris	mg/g WAT	713 ± 29	701 ± 62	852 ± 1	0.0440
tissue density	g/g WAT	0.9246 ± 0.0130	0.9208 ± 0.0129	0.9282 ± 0.0048	NS
fat density	g/mL WAT	0.922 ± 0.022			–
debris + interstitial space	µl/g WAT	237 ± 97	275 ± 92	145 ± 106	–

The data are the mean ± SD of different [N: PG = 2; MES = 3; SC = 4] 2-rat tissue pools. Statistical significance of the differences between groups (1-way anova). The column p represents the p values corresponding to the effect of WAT site. NS = p > 0.05

The number of RBC may indicate a rough approximation to the volume of blood vessels present in the tissue, despite exsanguination. Assuming a standard hematocrit value of 45%, the number of RBCs found in MES WAT represent about 17 µL of blood per g of fresh tissue, whilst the values for SC WAT and EPI WAT were 7.8 µL/g and 10.6 µL/g, respectively. The data agree with a higher blood irrigation of MES WAT compared with the other sites.^29^ Despite their different localization and function, MES and SC WAT seem more structurally closer than when compared with EPI WAT: a) the ratio of mean values for nucleated cell volumes (adipocytes/NSC) was 624 and 600, respectively, for MES and SC, but double, 1207 for EPI WAT. b) The ratio of nucleated cell numbers (NSC/adipocytes) was 13.7 for SC, 13.0 for MES and 9.6 for EPI. Nevertheless, the closeness of these data suggest that under standard (i.e. no inflammatory conditions, as is the case), the relationship of NSC and adipocyte numbers, despite their sizes, were rather uniform.

The proportion of fat in WAT agrees with the larger adipocyte size in the EPI site, which translates into significant differences in the proportion of tissue triacylglycerol (TAG) reserves. Curiously, this results in probably smaller (no statistical calculation was done because the data were derived from measurements obtained from transformed data) interstitial space for EPI WAT compared with the other two smaller-cell WAT sites. The proportion of tissue water (but also that of debris + interstitial space) was lowest in EPI WAT, which had the highest fat content, the reverse being true for SC WAT. However, the density of the tissue from different sites was practically the same and close to the actual density of fat (all measured at 22ºC).

### RBC lactate production

Since we could not isolate the RBCs from WAT collagenase digests, we simply counted their numbers, and then related their ability to metabolize glucose to results obtained using RBC taken from blood (or simply using blood as ‘suspension’ of RBCs). Since we assumed that all RBCs behaved equally irrespective of WAT site, we used the analysis of glucose consumption or lactate production simply to time of incubation, initial glucose and number of cells, containing a calculated value for lactate production in each WAT site sample.

**Figure 1.**
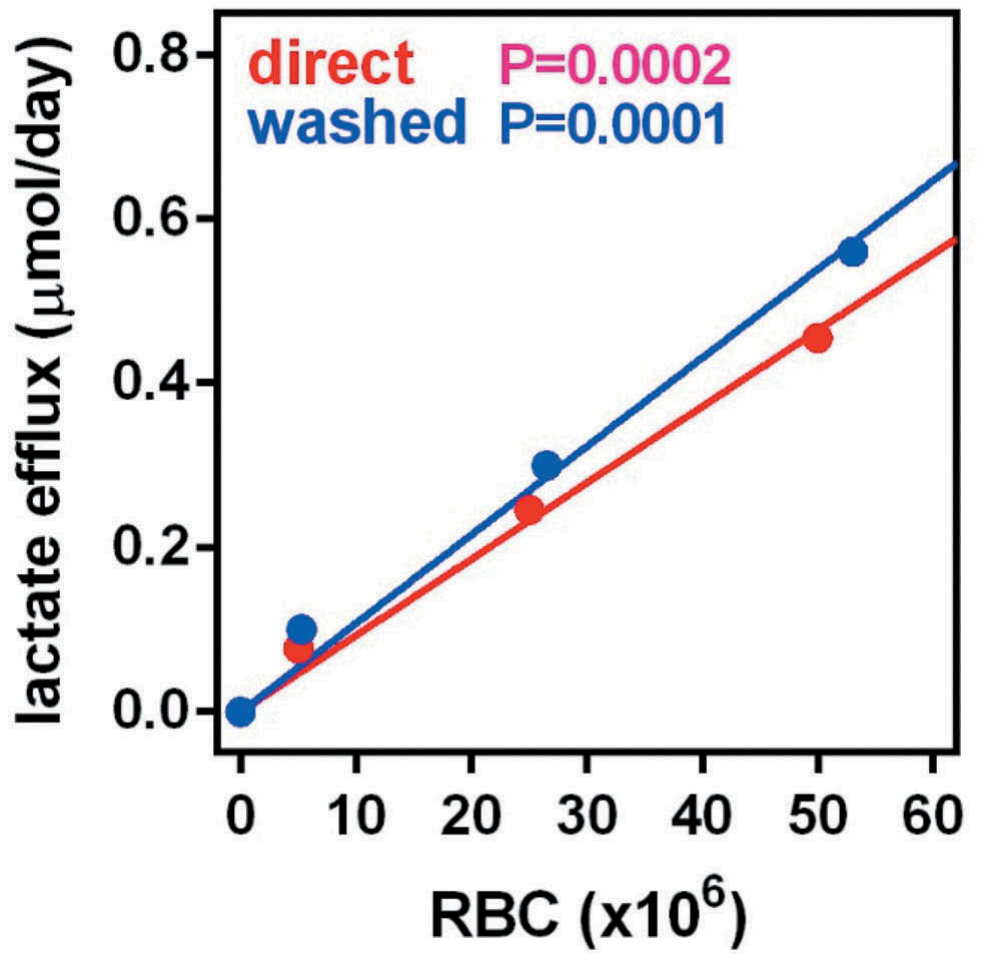
Production of lactate by RBCs incubated in medium with 14 mM glucose The data are the mean ± sem of 4 samples of adult rat blood. The blue dots and line coprrespond to samples of blood washed and processed in the same way than tissue extracts. Red dots and line are the results of direct incubation of whole fresh blood, The X axis represents the the number of RBC per well and the Y axis the amount of lactate released in micromoles per day. The p values represented in the Figure correspond to the statistical significance if the correlation between cell numbers and lactate efflux. No statistically significant differences were appreciated between the values for fresh blood and washed RBCs.

Figure 1 presents the production of lactate by fresh blood RBC incubated under the same conditions than the cells extracted from WAT. The number of RBC present in the actual stromal cell suspensions used were in a range comparable to the intermediate concentration of cells depicted here. The results obtained with washed cells were not significantly different, and are not shown. The production of lactate was marked and depended on incubation time and number of cells. The production of lactate was not significantly altered by glucose concentration in the 7/14 mM range studied (data not shown).

### Glucose uptake and metabolite efflux from incubated adipocytes and NSC

Samples from SC, MES and EPI WAT adipocytes and NSC were incubated for 24 h in 7 mM or 14 mM (initial values) of glucose. Lactate, glycerol and NEFA efflux were measured in the medium, as well as the disappearance of glucose, and used to determine the metabolic change per cell that will allow the estimation of global WAT capacity to use glucose.

Table 2 shows the glucose uptake and lactate, glycerol and NEFA efflux from incubated adipocytes, NSC and RBC. The values for NSC shown are the result of discounting the values of the incubated crude cell preparations minus the calculated contribution of RBCs. The only statistically significant difference between groups (taking as comparative factors site and initial medium glucose) was the effect of WAT site on glucose uptake, with maximal values in MES-WAT. No significant effects of glucose concentration were observed on lactate efflux of adipocytes or NSC. The lactate efflux of RBC was practically the equivalent of the glucose taken up, as expected. The relationships between glucose consumption and lactate efflux were closer to carbon equivalence in NSC than in adipocytes.

**Table 2.** WAT site cell glucose uptake and metabolite efflux of young male Wistar rats.

cell type & units (per cell and 24 h)		SC WAT		MES WAT		EPI WAT	
		7 mM	14mM	7 mM	14mM	7 mM	14mM
glucose uptake							
adipocytes	pmol	4.56 ± 0.68	4.94 ± 0.58	7.81 ± 0.68	8.73 ± 0.99	3.36 ± 0.52	4.02 ± 1.95
NSC	pmol	0.344 ± 0.085	0.63 ± 0.29	0.447 ± 0.077	0.811 ± 0.082	0,784 ± 0.150	1.06 ± 0.45
RBC	fmol	5.71 ± 1.70 [*]					
lactate efflux							
adipocytes	pmol	3.63 ± 0.56	3.90 ± 0.42	3.93 ± 0.91	3.88 ± 1.20	2.47 ± 0.48	2.81 ± 0.42
NSC	pmol	0.647 ± 0.214	0.504 ± 0.183	0.796 ± 0.141	0.876 ± 0.196	0.550 ± 0.222	0.564 ± 0.217
RBC	fmol	11.4 ± 3.4 [*]					
glycerol efflux							
adipocytes	pmol	1.56 ± 0.35	1.52 ± 0.35	2.26 ± 0.42	2.40 ± 0.33	1.71 ± 0.16	1.95 ± 0.21
NSC	fmol/	not detected					
NEFA efflux							
adipocytes	pmol	0.401 ± 0.109	0.385 ± 0.119	0,752 ± 0.407	0.360 ± 1.67	0.242 ± 0.038	0.178 ± 0.023
NSC	pmol	not detected					

The data are the mean ± sem of samples (N = 4; N = 3 for 14 mM glucose in MES and EPI), each corresponding to two pooled rat tissues; [*] calculated value (from data shown in Figure 1). Statistical significance of the differences between groups (2-way ANOVA). The only significant (p = 0.0008) relationship was that of glucose uptake by adipocytes with respect to WAT site.

The release of either glycerol or NEFA by NSC to the medium were negligible (small values, not significantly different from zero). The same can be said (as expected) for RBC.

**Figure 2.**
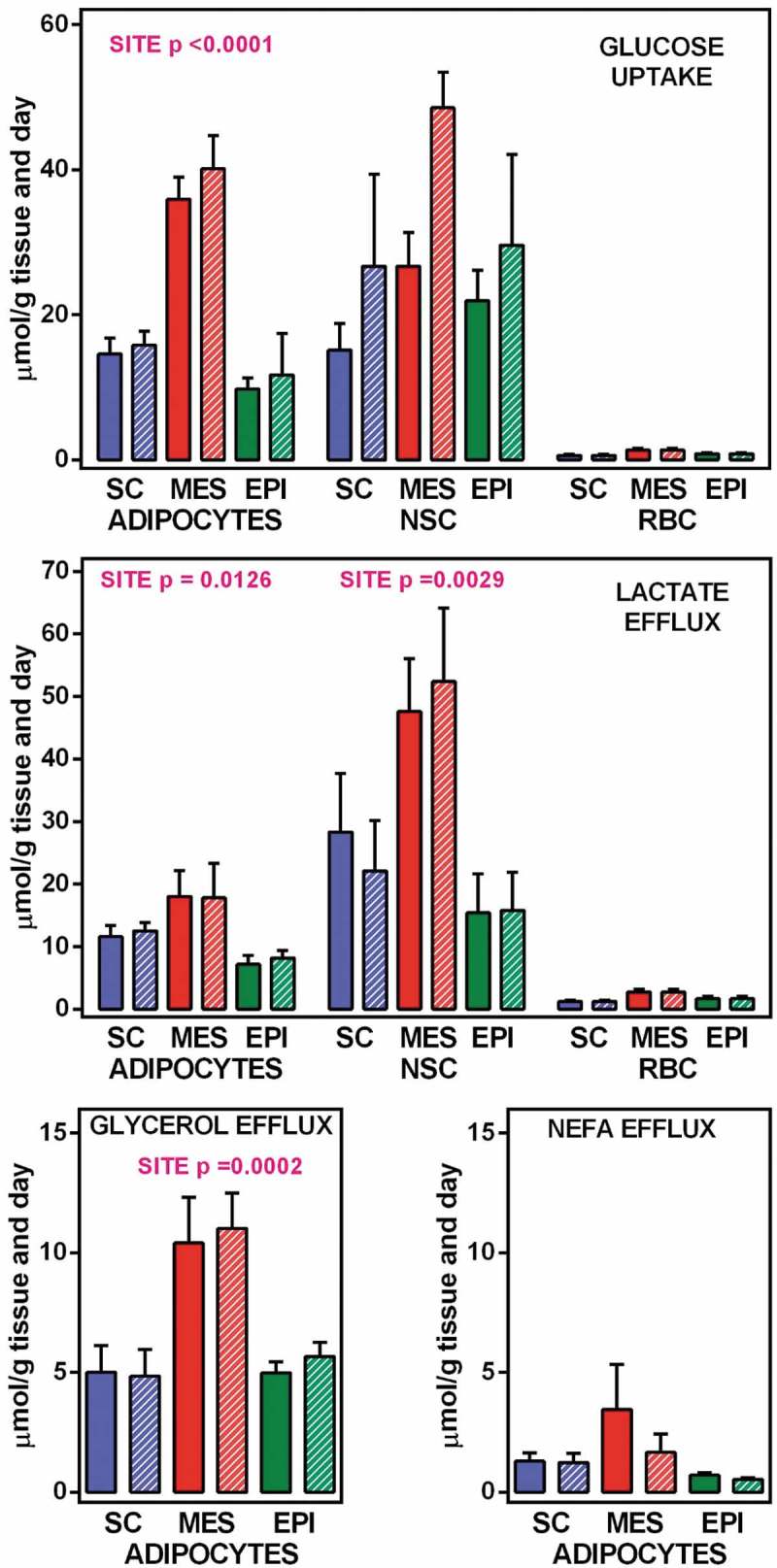
Glucose uptake, and lactate, glycerol and NEFA efflux from WAT component cells corresponding to 1 g of tissue SC = subcutaneous WAT (blue); MES = mesenteric WAT (red); EPI = epidydimal WAT (green). Full columns = 7 mM glucose, white-dashed columns = 14 mM glucose. The data are the mean ± sem of two-rat pooled samples (N = 4; N = 3 for 14 mM glucose in MES and EPI) obtained from adult male rats. The data for glucose uptake and lactate efflux correspond to the number of adipocytes, NSC and RBC contained in 1 g of tissue (Table 1) multiplied by their individual uptake/efflux data (Table 2). The data for glycerol and NEFA correspond exclusively to the adipocytes present in 1 g of tissue, since no efflux of either was observed in NSC or RBC. Statistical significance of the differences between groups (2-way ANOVA): the data for site in adipocytes and NSC are indicated in the Figure itself, the absence of data indicates that the differences, if any, were not significant (P > 0.05); no significant differences were observed for the effect of initial glucose concentration.

When the individual cell data were adjusted by the number of cells of each group, in order to show the composite estimated values corresponding to all cells present in 1 g of WAT, we obtained the results depicted in Figure 2. These data reflect a minimal quantitative importance of the RBC fraction both for glucose uptake and lactate efflux. Consequently, all further comparisons were limited to NSC and adipocytes. With respect to glucose uptake, as well as lactate and glycerol efflux, adipocytes showed a clear effect of site, with higher values in MES WAT. This effect was also significant for lactate efflux on NSC, with, again, a higher production of lactate in MES WAT. No significant changes were observed in NEFA efflux (only different from zero in adipocytes). However, glycerol efflux rates were different for the sites studied, the highest values being those of MES adipocytes.

Taken as a whole, and contrary to what we expected, in all three sites, lactate efflux by NSC was higher than that produced by the adipocytes. It is remarkable to add, that there were no statistically significant effects of initial glucose concentration on any of the parameters analyzed.

### Comparative gene expression in WAT sites

After incubation, adipocytes and NSC suspensions were used to extract their RNA, which was used to analyze the expression of a number of glycolytic and lipogenic enzymes, in the different WAT sites studied. The number of copies per cell in each cell fraction was then used to calculate the global ‘power’ of expressed proteins in adipocytes and NSC of the three WAT sites analyzed.

Table 3 presents the number of copies per cell of the specific mRNA for genes related to the production of 3C substrates from glucose. All genes, except *Pdk4* and adipocyte *Hsl* and *Lpl* showed a significant effect of site in their expression. In general, the highest values were found in MES WAT

**Table 3.** WAT site expression (in specific mRNA copies per cell) of genes related with glycolysis and glycerol metabolism of young male Wistar rats.

gene		cells	SC WAT		MES WAT		EPI WAT		p	
			7 mM	14mM	7 mM	14mM	7 mM	14mM	site	glucose
*Glut1*	glucose transporter 1	adipocytes	75.7 ± 5.0	128 ± 14*	304 ± 56*	287 ± 61	111 ± 11	123 ± 22	<0.0001	NS
		NCS	20.0 ± 4.8	26.3 ± 9.2*	31.1 ± 4.3*	52.6 ± 5.6*	16.3 ± 5.6*	23.3 ± 5.5	0.0050	0.0322
*Hk1*	hexokinase 1	adipocytes	172 ± 15	222 ± 29*	488 ± 52*	500 ± 35*	343 ± 54	394 ± 108	0.0011	NS
		NCS	11.9 ± 2.6	6.71 ± 4.04	26.0 ± 1.6	32.4 ± 0.1*	18.8 ± 6.7	20.5 ± 4.7	0.0007	NS
*Ldha*	type a lactate dehydrogenase	adipocytes	1003 ± 196	1274 ± 202*	2271 ± 260*	1963 ± 214	1959 ± 157	1988 ± 154	0.0003	NS
		NCS	95.6 ± 11.6	77.8 ± 11.8*	222 ± 9*	265 ± 38*	189 ± 20	168 ± 13	<0.0001	NS
*Ldhb*	type b lactate dehydrogenase	adipocytes	164 ± 21	176 ± 8*	332 ± 62*	362 ± 51*	339 ± 32	340 ± 23	0.0003	NS
		NCS	1.15 ± 0.18	0.66 ± 0.37*	3.63 ± 0.48*	1.96 ± 0.69*	3.94 ± 0.48	3.66 ± 0.32	<0.0001	0.0345
*Pfkl*	phospho-fructo-kinase liver	adipocytes	131 ± 11	195 ± 24	392 ± 73*	404 ± 24*	170 ± 29	182 ± 46	<0.0001	NS
		NCS	15.1 ± 2.6	7.56 ± 3.24	34.1 ± 1.2	41.2 ± 7.3*	17.2 ± 4.8	16.9 ± 3.3	<0.0001	NS
*Phgdh*	3-phospho-glycerate DH	adipocytes	17.5 ± 1.5	25.5 ± 3.7*	52.1 ± 11.6*	58.0 ± 9.8*	42.3 ± 4.7	44.0 ± 9.2	0.0013	NS
		NCS	2,64 ± 0.33	2.44 ± 0.53*	7.23 ± 0.96	8.14 ± 0.81*	5.40 ± 1.46	5.67 ± 1.60	0.0013	NS
*Gpam*	glycerol-3P acyl-transferase	adipocytes	15.6 ± 1.1	19.3 ± 4.7*	58.3 ± 10.7*	54.7 ± 15.6	38.8 ± 2.4*	34.7 ± 10.7*	0.0018	NS
		NCS	*0.478 ± 0.100*	0.439 ± 0.115*	*1.12 ± 0.25*	1.32 ± 0.54*	0.982 ± 0.137	0.904 ± 0.136	0.0298	NS
*Pdk4*	pyruvate DH kinase 4	adipocytes	4.76 ± 2.36	5.16 ± 2.52*	19.9 ± 15.9*	9.50 ± 1.81*	2.83 ± 0.92	2.72 ± 0.85	NS	NS
		NCS	0.061 ± 0.007*	0.024 ± 0.024*	*0.050 ± 0.016*	0.047 ± 0.002*	0.063 ± 0.009*	0.013 ± 0.003*	NS	NS
*Fas*	fatty acid synthase	adipocytes	97.2 ± 6.3	191 ± 30*	302 ± 54*	478 ± 84*	181 ± 27	241 ± 63	0.0004	0.0166
		NCS	5.42 ± 0.83	3.49 ± 0.99*	11.2 ± 1.8	19.7 ± 5.1*	8.62 ± 1.35	10.4 ± 1.9	0.0012	NS
*Hsl*	hormone-sensitive lipase	adipocytes	45.0 ± 5.5	38.4 ± 5.5*	48.6 ± 8.6*	48.4 ± 6.5	98.3 ± 34.1	109 ± 54	NS	NS
		NCS	*0.739 ± 0.282*	*0.364 ± 0.079*	*0.966 ± 0.182*	1.01 ± 0.15*	0.566 ± 0.050*	*0.599 ± 0.011*	0.0195	NS
*Atgl*	adipose TAG lipase	adipocytes	218 ± 20	320 ± 45	488 ± 65*	560 ± 56*	547 ± 71	465 ± 74*	0.0005	NS
		NCS	*5.69 ± 0.85*	5.22 ± 0.67*	11.8 ± 2.8	18.4 ± 1.03*	6.08 ± 1.48	6.08 ± 0.92	<0.0001	NS
*Lpl*	lipoprotein lipase	adipocytes	859 ± 42	1106 ± 228*	1437 ± 222*	1537 ± 238*	1466 ± 262	1681 ± 358	NS	NS
		stromal	2.62 ± 0.87	0.89 ± 0.20*	3.50 ± 0.38	3.51 ± 1.37*	3.32 ± 0.42	3.53 ± 0.26	0.0412	NS

The data are the mean ± sem of N = 4 different (* N = 3) two- rat pooled samples. Data in italics were obtained with 30 or more cycles. Statistical significance of the differences between groups (2-way ANOVA). The far right columns show the p values for site and initial glucose concentration. NS = p > 0.05

**Table 4.** List of primers used.

gene	protein	direction	sequences	bp
*Glut-1*	glucose transporter type 1, erythrocyte/brain	5ʹ >	GCTCGGGTATCGTCAACACG	97
		> 3’	ATGCCAGCCAGACCAATGAG	
*Hk1*	hexokinase type 1	5ʹ >	TGGATGGGACGCTCTACAAA	100
		> 3’	GACAGGAGGAAGGACACGGTA	
*Ldha*	L-lactate dehydrogenase a	5ʹ >	AAAGGCTGGGAGTTCATCCA	96
		> 3’	CGGCGACATTCACACCACT	
*Ldhb*	L-lactate dehydrogenase b	5ʹ >	GCGAGAACTGGAAGGAGGTG	145
		> 3’	GGGTGAATCCGAGAGAGGTTT	
*Pfkl*	phospho-fructokinase, liver, b-type	5ʹ >	CAGCCACCATCAGCAACAAT	90
		> 3’	TGCGGTCACAACTCTCCATT	
*Pfkm*	phospho-fructokinase, muscle	5ʹ >	CATCCCATTTGTGGTCATTCC	149
		> 3’	TAAACACTCGCCGCTTGGT	
*Phgdh*	phospho-glycerate dehydrogenase	5ʹ >	CTGAACGGGAAGACACTGGGAA	138
		> 3’	AACACCAAAGGAGGCAGCGA	
*Gpam*	glycerol-3-phosphate acyl-transferase, mitochondrial	5ʹ >	GGTGAGGAGCAGCGTGATT	129
		> 3’	GTGGACAAAGATGGCAGCAG	
*Pdk4*	pyruvate dehydrogenase kinase, isoenzyme 4	5ʹ >	CTGCTCCAACGCCTGTGAT	142
		> 3’	GCATCTGTCCCATAGCCTGA	
*Fas*	fatty acid synthase	5ʹ >	CCCGTTGGAGGTGTCTTCA	117
		> 3’	AAGGTTCAGGGTGCCATTGT	
*Hsl*	lipase, hormone sensitive	5ʹ >	TCCTCTGCTTCTCCCTCTCG	108
		> 3’	ATGGTCCTCCGTCTCTGTCC	
*Atgl*	adipose triacylglycerol lipase	5ʹ >	CACCAACACCAGCATCCAAT	120
		> 3’	CGAAGTCCATCTCGGTAGCC	
*Lpl*	lipoprotein lipase	5ʹ >	TGGCGTGGCAGGAAGTCT	116
		> 3’	CCGCATCATCAGGAGAAAGG	
*Arbp*	0S acidic ribosomal phospho-protein PO [housekeeping gene]	5ʹ >	CCTTCTCCTTCGGGCTGAT	122
		> 3’	CACATTGCGGACACCCTCTA	

The effects of glucose were much more limited than those of site, the only changes observed were in NSC *Glut1* and *Ldhb* of NSC, and in adipocyte *Fas*. No other effect was observed, and those listed were close to the limit of significance. Some of the data presented may be partially artefactual, because of the low number of copies counted, in some cases lower than 1 per cell. This is probably a consequence of the NSC being a complex mixture of quite different types of cells, a condition magnified in this case by the different proportions of NSC found in different sites per unit of tissue mass. The lowest values of copies per cell in NSC were for genes related to lipid metabolism. (*Gpam, Hsl, Fas, Atgl* and *Lpl*), but included a critical regulatory enzyme *Pdk4* and the lactate dehydrogenase isozyme *Ldhb*, much more abundant in adipocytes. These data suggest a probable different regulation of lactate dehydrogenase in adipocytes and the NSC as a whole.

**Figure 3.**
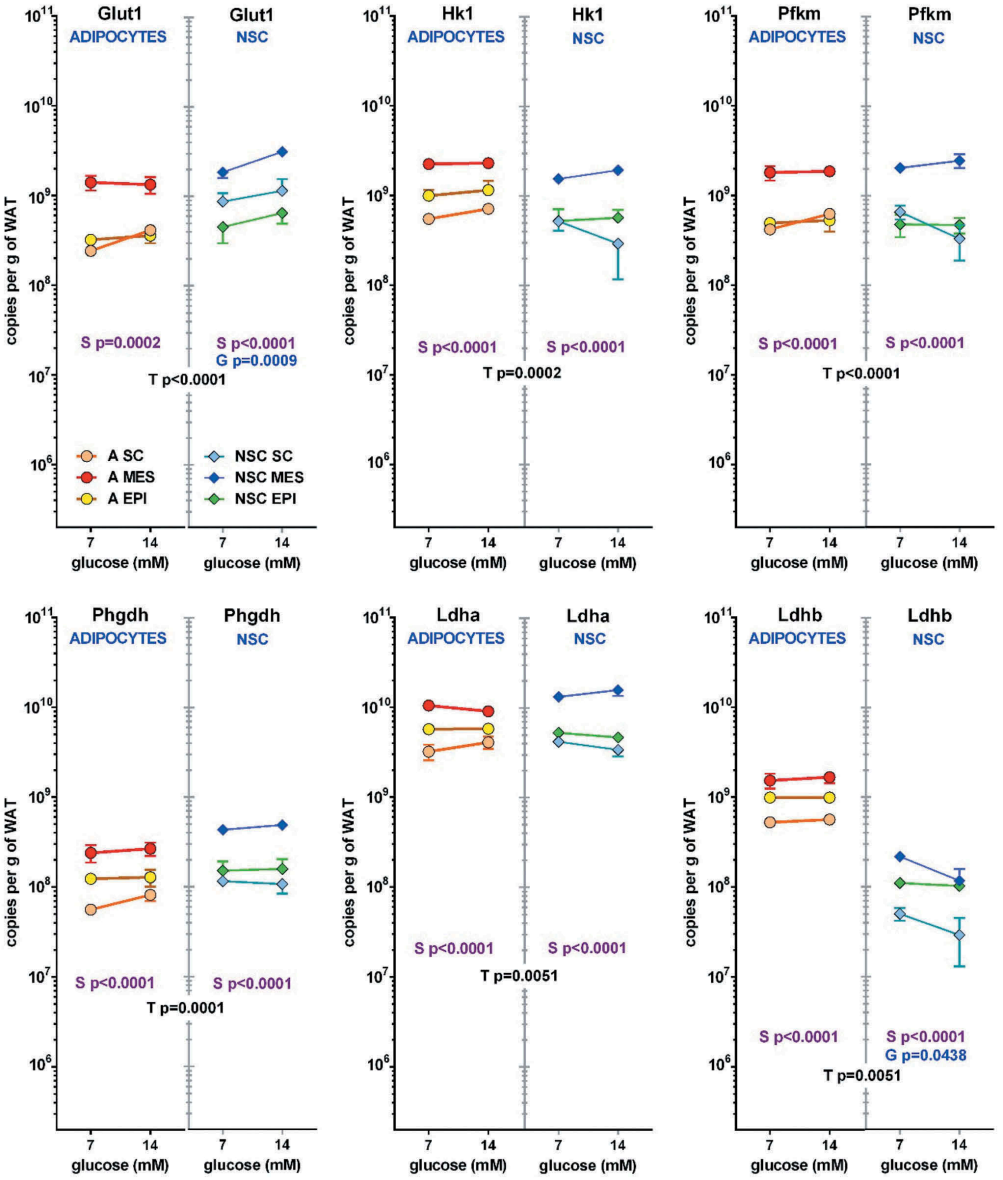
Number of copies of glycolysis-related genes in adipocytes and NSC contained in 1 g of WAT from different sites (SC, MES and EPI) or male adult rats The data correspond to the mean ± sem of two-rat pooled samples (N = 4 or N = 3 in the groups indicated in Table 3). The data have been presented in a of magnitude logarithmic scale (with more than five orders of magnitude span) for easy direct comparison of the abundance of all gene transcripts. The values shown were calculated from the data presented in Tables 1 and 3. Dot and line colours are represented in the Figure: Adipocytes: orange A SC, red A MES, yellow A EPI; NSC: light blue NSC SC, blue NSC-EPI, green NSC-EPI., Statistical significance of the differences between groups (3-way ANOVA). T: corresponds to differences between the ‘type’ or cell (adipocyte vs. NSC), in black, S: refers to the differences between ‘sites’ (SC, MES, EPI) within the same cell group, in purple, and G represents the statistically significant differences in expression induced by the initial ‘glucose’ concentrations, in blue.

**Figure 4.**
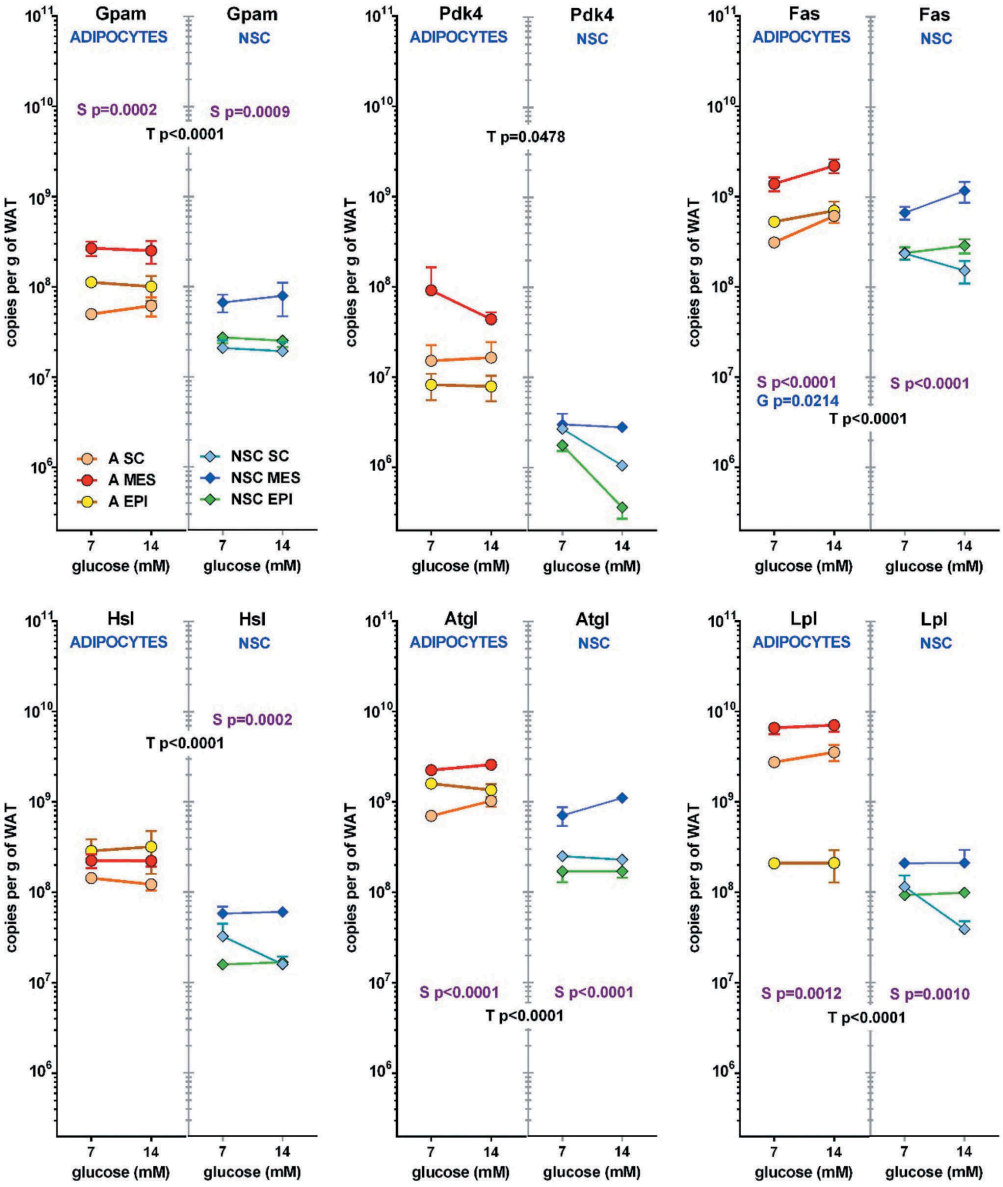
Number of copies of glycerol and lipid metabolism-related genes in adipocytes and NSC contained in 1 g of WAT from different sites (SC, MES and EPI) or male adult rats The data correspond to the mean ± sem of 4 different two-pooled rat samples. All conventions are the same described for Figure 3.

In a way parallel to the proportional representation of cells’ effects on medium metabolites, Figures 3 and 4 show the contribution of adipocytes and NSC to the total number of copies for the genes studied per g of WAT. The contribution of the low number of adipocytes compared with the NSC were similar for most glucose-lactate metabolism-related genes (Figure 3). In the six genes depicted: *Glut1, Hk1, Pfkm, Phgdh, Ldha* and *Ldhb*, the effect of ‘type of cell (NSC or adipocyte) was statistically significant, and in all cases and in both cell types the effect of site was also significant. In addition, in NSC, *Glut 1* and *Ldhb* showed a significant effect of glucose. In most cases MES WAT showed the highest contributions (and SC the lowest) both in adipocytes and NSC, with global gene expression values markedly parallel in all cases except for lactate dehydrogenase b (*Ldhb*), which expression was much lower than that of the a isozyme (*Ldha*), showing even lower values for NSC compared with adipocytes. This case being the only ‘discordance’ in that series of data.

The trend shown by *Ldhb* was partially repeated (Figure 4) in *Gpam, Pdk4, Hsl, Atgl*, and *Lpl*, which represent a general, albeit limited, view of lipid metabolism. In all genes studied, the effect of type of cell was, again, fairly patent, this being in part a consequence of different cell size, even allowing for the inert vacuole mass of TAG. The effects of site were significant for *Gpam, Fas, Atgl* and *Lpl*, for both cell types, as well as *Hsl* in NSC. In adipocytes, the effect of initial glucose concentration was observed only in *Fas*. The gene expression values per unit of tissue weight were clearly higher in adipocytes than in NSC, one-to-two orders of magnitude lower. The primacy of MES was maintained, but the differences between SC and EPI WAT were less marked, interchanging positions, in clear divergence with the glycolytic data of Figure 3. In adipocytes, *Lpl* expression in EPI WAT was more than one order of magnitude lower than in SC and MES. The expression of *Pdk4*, normally low, was even lower in NSC, tending to decrease with glucose concentration (the results were not significant), which tend to agree with the reverse trend on *Fas*, since lipogenesis requires the formation of acetyl-CoA and the decrease in *Pdk4* hints at a lower inhibition of pyruvate dehydrogenase.

## Discussion

In WAT, the global importance of NSC has been widely recognized, especially its implication in questions of regulation,^30^ defense,^31^ regeneration^32^ and even metabolic control^33^ via hormones and cytokines. However, and despite its also accepted variety and diversity depending on WAT site, to our knowledge, no actual analysis of its overall metabolic contribution to the handling of substrates has been so far analyzed nor established. The results presented here are only the tip of an iceberg that needs to be acknowledged, measured, known and its true magnitude ascertained.

The heterogeneous structure of WAT prevented a direct *in vivo* approach. Consequently, we had to introduce an initial tissue fractioning step: separating adipocytes from the rest of WAT cells, and analyzing their capacity to use glucose and yield 3-carbon substrates. We are aware that this is only an approximation to the *in vivo* situation, since cells in primary culture do not behave in the same way than incorporated in a stromal organization within a living tissue.

We are aware that the methodology we used should be refined further to gain sufficient insight on the quantitative role of NSC in substrate handling, but the basic concepts have been already established,^26^ and the isolation, incubation, viability and analyses needed are available, often being standard and commonly used. The data presented here continues what has been developed in a series of preceding methodological and quantitative studies that drove us to the findings shown here.^18-20,26-29,34^

The deconstruction-isolated cell- analysis-reconstructive computing of results is not a substitute for the *in vivo* (unfeasible) analysis, but at least may provide an approximate idea of the metabolic potential of this widely diverse group of cells. The data presented, support the idea that WAT’s own metabolism is even more complex than usually assumed. Adipocytes represent less than 10% of nucleated cells, despite each containing about 10-fold the ‘live cell’ volume of NSC, overall, a few microliters per gram of WAT.^28^ It has been assumed that WAT reason to exist is essentially defined by adipocytes and their TAG stores,^35^ metabolic activity being centered on lipogenesis from glucose and lipolysis of TAG. This may be largely true for the largest and more deposit-prone sites, such as EPI WAT, but not as much for the more metabolically active MES WAT^36^ or the smaller control-related deposits such as perivascular^37^ or pericardic^38^ depots. However, and with respect to glycolysis to lactate our data show that NSC glycolytic activity was higher than that of adipocytes in all three sites studied. Nevertheless, the difference was even higher for the most active WAT, MES.

The data obtained for the NSC almost complete glycolytic conversion of glucose to lactate strongly support their anaerobic orientation, even in the presence of abundant oxygen. In this case, both NSC and adipocytes (and even RBCs) tend to convert glucose to 3C-units, largely lactate, responsible for a large share of rodent cell energy supply^39^

The question is to know whether the observed glycolytic function is intrinsic or adopted. It may be postulated that the presence of large adipocytes, in which most of their cytoplasm was just a thin layer between the plasma membrane and the fat vacuole, too far away from mitochondria^27^ for efficient oxidative metabolism and lipogenesis to take place;^26^ and thus becoming, necessarily, glycolytic,^40^ despite having a sufficient availability of oxygen. There is a long list of WAT NSC types, and their dependence on oxygen is patent when studied from other sources.^41^ Thus, it is difficult to attribute this WAT-inbred trend simply to an adaptation to relative anaerobiosis, in part due to restricted blood flow (oxygen supply) to the tissue,^21^ but more to the ability to survive with the limited amount of ATP that glycolysis provides. The high lactate production was unrelated to high medium oxygen, in a parallel way to that shown by adipocytes.^27^*^,^*^42^ This relative independence with respect to oxygen agrees with the limited WAT oxygen consumption *in vivo*^21^ and supports massive energy-inefficient use of glucose for glycolysis to lactate instead of its complete oxidation

In studies of non-adipocyte WAT cells, lactate production has been often associated to hypoxia.^43^ The results presented here complicate the widely acknowledged relationship of hypoxia in WAT with the negative consequences of inflammation.^44^ How can hypoxia become a key factor eliciting a pathologic inflammatory response in a tissue which ‘main’ cells, adipocytes, live off the ATP generated by glycolysis,^19,27^ limiting oxygen use largely for lipogenesis but not for energy^45^ ? Now, we have found that the ‘other’ WAT cells, NSC, a diverse mixture of structural, epithelial, immune system cells, stem cells, fibroblasts and others, may thrive, as a whole, also using the energy inefficient glycolytic pathway instead of the more efficient mitochondrial oxidative metabolism. In addition, these small cells do not have the geometric constrictions of adipocytes (distance of most zones of cytopkasm to mitochondria). These data clearly hint at a “whole WAT” adaptation to low oxygen and/or the purpose to convert excess glucose to 3C fragments, which runs in parallel for adipocytes and NSC. This trend is maintained even when oxygen is widely available (but glucose is available too), and may represent a true distinguishing WAT characteristic.

Regulation of WAT blood supply has been postulated as a mechanism of defense against excess substrate availability;^12^ but it reduces too the oxygen supply, which may result in hypoxia, even counting with the Bohr effect, which facilitates the release of oxygen from RBC when lactate is high.^46^ Probably, the adaptation to low oxygen renders blood oxygen supply as secondary for WAT (few cells, low intrinsic needs) leaving blood flow control as – essentially – a regulatory factor for substrate supply.^47^ Our data, presented here also hint to MES WAT having a higher blood irrigation^48^ than the other sites, but – again – lactate production is highest in that site.

The gearing of adipocytes and NSC for glycolysis results fully apparent in Figure 3, with a marked parallelism on the quantitative expression of these two ‘halves’ of active WAT: adipocytes and NSC. A number of differences (lipid metabolism, lactate dehydrogenase ratio) show their different origin and function; but the fact that both participate of WAT glycolytic activity raises the question of the metabolic importance of these cells with respect to whole-body energy handling. It must be taken into account that the mass of the adipose organ is one of the largest for any organ or system in the body.

The capability to thrive with low oxygen may be of paramount importance for a tissue implicated in tasks of regeneration and defense, being able to grow in hypoxic niches where angiogenesis is a critical tool for repair and normalization.^49^ The parallelism of this facet of WAT and tumors ends here, since the density of substrate and oxygen consumption per unit of weight are quite different, as are the rates of glucose consumption (i.e. the Warburg Effect^50^) and the unique property of WAT to produce large amounts of glycerol alone, without the concurrence of NEFA excretion.^26^ Here we have found that this is a peculiarity of adipocytes, since NSC did not release either glycerol or NEFA to the medium.

Glycerol levels and uptake are not as strictly regulated as is glucose by insulin, thus WAT breaking up of glucose to 3C units has the advantage to eliminate excess glucose (and thus lower glycemia)^51^ without creating a problem of energy supply to the brain, which can be sustained by both lactate and glycerol.^52^*^,^*^53^

In sum, we found that NSC can match the glycolytic activity of adipocytes, being responsible for a significant portion (which may be a major part) of glycolytic conversion of blood glucose to lactate, a main substrate used to sustain a sizeable part of the energy needs of most body cells.^39^ However, the efflux and gene expression data suggest that NSC do not participate in WAT active glycerol metabolism. Thus, we also conclude that glycerol is the exclusive product of adipocytes, and postulate that its fate is probably to provide energy to other tissues, with, perhaps, a more direct relationship with the brain, avid consumer of this polyol^53^ and lactate^54^ for energy. There are marked differences between WAT sites, with mesenteric WAT playing a more active role, probably related to its location besides the intestine, for glucose disposal and glycerogenesis.

## Methods

### Rats and sampling

All animal handling procedures and the experimental setup were in accordance with the animal handling guidelines of the corresponding European and Catalan Authorities. The Committee on Animal Experimentation of the University of Barcelona specifically authorized the procedures used in the present study.

Adult male Wistar rats (Janvier, Le Genest-Saint Isle, France), aged 11 weeks when killed, were used. The animals weighed 426 ± 12 g and were kept in two-rat cages with wood shards as bedding material, at 21-22ºC, and 50–60% relative humidity; lights were on from 08:00 to 20:00. They had unrestricted access to water and standard rat chow (#2014, Teklad Diets, Madison, WI USA).

The rats (N = 12) were killed, under isoflurane anesthesia, by exsanguination from the exposed aorta. They were dissected, and samples of epididymal (EP), mesenteric (MES) and subcutaneous (SC; both inguinal cordons) WAT were extracted. In order to obtain sufficient material, same weight samples from the same-site of two size-matched rats were pooled, minced with scissors and thoroughly mixed before further processing.

### WAT cell isolation and preparations

Samples of whole WAT were reserved and used for lipid and water content analysis as well as for density estimation as previously described.^28^ Cells were isolated^55^ at 37ºC for 1 h in a shaking bath using collagenase (LS004196, type I, from Worthington Biomedical, Lakewood NJ USA) in 2.5 volumes of modified Krebs-Henseleit buffer. Then, the cell suspension was filtered through a double layer of nylon hose. The retained debris was recovered and weighed. The cell suspension was transferred to vertical syringes and left standing for 5–6 minutes at room temperature. Adipocytes formed an upper layer, floating over a liquid phase. The latter was slowly drained from the syringe and the upper adipocyte mass was left in it as previously described. The adipocyte layer was gently suspended again in fresh incubation buffer (free of collagenase) and the process of mixing and draining was repeated twice, discarding the washing fluids.^49^ Aliquots of the adipocyte layer were used for cell size estimation, lipid content (for analysis of recovery), and for incubation as described below.^26^

The first washing contained most of the non-attached stromal cells; it was filtered through a 100 µm mesh to remove debris. The filtrate was centrifuged 8 min at 500 × g. The NSC were then re-suspended, and used directly for incubations, cell number estimation and analysis of red blood cell content, as previously described.^28^ All cell preparations were maintained at room temperature (c. 22ºC), and manipulated for a time as short as possible; adipocytes were used immediately after the final washing.

The stromal cell space in the isolated cell suspension was used to relate their numbers and volumes to initial tissue weight; it was estimated as the sum of the volume of the lower phase of adipocyte separation (extracted in the syringe), plus a part of the volume of the adipocyte phase not occupied by the adipocytes themselves. This latter volume was the difference between the volume of the phase and that of adipocytes, calculated from their numbers and cell volumes.^28^ Obviously, the first separation of adipocytes and stromal cells left a high number of the latter mixed with adipocytes. The three successive washings resulted in the presence (calculated) of, at most, 0.1% of the initial free stromal cells in the final washed adipocyte fraction (down from an initial 7.3%). This assumption does not take into account stromal cells bound, retained or attached to the larger adipocytes.

### Measurement of isolated cell parameters

Adipocyte suspensions were examined using a Neubauer chamber (#717,810 Neubauer improved bright line, Brand Gmbh, Wertheim, Germany) following the procedure already described by us.^28^ At least 16 fields for sample were photographed using an inverted microscope. Cells were identified, counted, and their size analyzed (under the conditions used, all cells adopted a spheroid form), using the FIJI ImageJ software (http://imagej.nih.gov/ij/).^56^

Non.adipocyte stromal cells (i.e. including RBCs) were analyzed in each sample with the Scepter 2.0 cell counter (EDM Millipore Corp, Billerica, MA USA) hand-held cell sizer, using two different cell-range tips for the Scepter: Sensor 40, for 3–18 µm particles’ size (PHCC40050, Merck Millipore, Darmstadt, Germany) and Sensor 60, for 6–36 µm particles’ size (PHCC60050, Merck MIllipore). RBCs were estimated from the counting of particles with volumes between 25 fL and 60 fL, since these limits included 90% of total RBC (unpublished data). The larger particles were considered to be nucleated stromal cells (NSC), as previously described.^28^ Particles smaller than 25 fL were also counted; they were considered to be, essentially, fat droplets and other small cell or fiber agglomerate debris.

### Cell incubation procedures

The complete procedure has been described previously by us;*^26^*^,^*^28^^,^*^29^ in short: Cell incubations were carried out using 12-well plates (#734-2324VWR International BVBA/Sprl., Leuven Belgium) filled with 1.7 ml of DMEM (#11966-DMEM-no glucose; Gibco, Thermo-Fisher Scientific, Waltham MA USA), supplemented with 30 mL/L fetal bovine serum (FBS, Gibco). The medium also contained 25 mM hepes (Sigma-Aldrich), 2mM glutamine (Lonza Biowhittaker, Radnor, PA USA), 1 mM pyruvate (Gibco), 30 mg/mL delipidated bovine serum albumin (Millipore Calbiochem, MA USA) and 100 nM adenosine, 100 U/mL penicillin plus 100 mg/L streptomycin (both from Sigma-Aldrich). The cells were supplemented with glucose (Sigma-Aldrich) to a final concentration of either 7 mM or 14 mM.

Each well received 400 µL of the corresponding cell suspension; after initial sampling, the final incubation volume was 2.0 mL. The cells were incubated at 37°C in a chamber ventilated with air supplemented with CO2 (5%), which gave a theoretical dissolved pO2 of 20 kPa.^19^ The cells were incubated for 24 h without any further intervention. Then, the wells’ contents were transferred with a pipette to small polypropylene tubes, which, in the case of adipocytes, were left standing for 5 min to pipette out the infranatant and immediately use *in situ* the adipocyte fraction for RNA extraction. In the case of NSC (containing the original tissue RBCs), the tubes were centrifuged for 8 min at 500 × g. Supernatant medium was extracted, and the cell precipitates (RBC and NSC) were immediately used for RNA extraction. All supernatants were aliquoted, frozen and stored at −20ºC until processed. The gene expression data was analyzed only in the 24 h samples, after incubation and harvesting, and were used to compare the different effects of exposure to glucose concentration. No time zero expression data (i.e. basal state, equivalent to that of initial WAT) were available, since the incubation started with cells just isolated after collagenase incubation and separation, and thus not kept under uniform conditions, and far away from those in the tissue. After one day of incubation, the cells had time to establish a higher degree of gene expression uniformity (with glucose, oxygen and other nutrients available) in which the only different variable was the initial glucose concentration.

### Analysis of metabolites in the medium

Medium glucose was measured using a glucose oxidase-peroxidase kit (#11,504, Biosystems, Barcelona Spain) to which we added 740 nkat/mL mutarrotase (porcine kidney, 136A5000, Calzyme, St Louis, MO USA).^57^ Lactate was measured with kit 1,001,330 (Spinreact, Sant Esteve d’en Bas, Spain); glycerol was estimated with kit #F6428 (Sigma-Aldrich). NEFA were measured using kit NEFA-HR (Wako Life Sciences, Mountain View, CA USA). Data for medium metabolites was referred to the number of cells in the well.

### Estimation of RBC lactate production under the conditions of incubation

The variable presence (despite the rats being exsanguinated) of RBC in the samples (affecting cell counts), and their glycolytic nature (affecting glucose uptake and lactate output) were possible sources of interference for the analysis of non-adipocyte production of lactate. Since no practical method was available to remove RBCs from the non-adipocyte cell preparations without producing unknown damage or modification to the other cells, we opted for the estimation of the eventual capability of RBCs to produce lactate using directly RBCs (from blood) alone. Two different preparations of RBCs were obtained from the pooled fresh (heparinized) blood of two ‘WAT donors’. In one, blood was suspended (diluted) in WAT cell isolation medium; then, the cells were centrifuged and washed in incubation medium and re-suspended (i.e. treated as cells isolated from WAT). This suspension was compared with the direct dilution of blood in incubation medium. Using the Scepter cell counter as described above, the number of cells in both suspensions were counted. Three cell concentrations were prepared: 5, 25 and 50 million cells per well. The RBCs were then incubated under the same conditions than the other cells for up to 48 h. The cells were harvested and discarded, and the media lactate was measured using the procedure described above. The glucose (and lactate) present in the RBC carried in the NSC fraction was at least one order of magnitude below the attomol/cell range; consequently, its eventual influence on the zero-time measurements of lactate and glucose was practically unmeasurable, i.e. negligible

### Gene expression analysis

Total cell RNA was extracted from all the harvested cells using the Tripure reagent (Roche Applied Science, Indianapolis IN USA). RNA content was quantified in a ND-1000 spectrophotometer (Nanodrop Technologies, Wilmington DE USA). RNA samples were reverse transcribed using oligo-dT primers (Gene Link, Westchester, NY USA) and the MMLV reverse transcriptase (Promega, Madison, WI USA) system.

Real-time PCR (RT-PCR) amplification was carried out using 10 μL amplification mixtures containing Power SYBR Green PCR Master Mix (Applied Biosystems, Foster City, CA USA), 4 ng of reverse-transcribed RNA and 150 nmol of primers. Reactions were run on an ABI PRISM 7900 HT detection system (Applied Biosystems) using a fluorescent threshold manually set to 0.5 for all runs.

A semi-quantitative approach for the estimation of the concentration of specific gene mRNAs per unit of tissue weight was used.^58^ *Arbp* was the charge control gene.^59^ We initially expressed the data as the number of transcript copies per nucleated cell.

The genes analyzed and a list of primers used are presented in Table 4.

### Data presentation and statistical procedures

Since a main objective of the study was to evaluate the eventual contribution of non-adipocyte cells to the efflux of glycolytic and lipolytic efflux, we presented the data in two complementary forms. The direct values of substrate efflux (uptake for glucose) and number of copies of specific protein mRNAs were presented per cell (in Tables), whereas, these same values corrected by the number of cells present in each tissue cell fraction per unit of whole tissue weight were shown in Figures. This way, in a theoretically reconstituted WAT each cell type: adipocytes, NSC and RBC showed its global comparative effect on each of the parameters measured, making approximate comparisons possible. This presentation also partly circumvented the problem of statistical comparisons of composite data (Figures), since the statistical power of Table data comparisons was more reliable despite the limited number of samples used.

Statistical analyses and the establishment of significant differences between groups (one- and two-way ANOVAs) were done with the Statgraphics Centurion XVI program (Statpoint Technologies, The Plains, VA USA).
